# Successful Coronary Artery Bypass Grafting in a Moyamoya Patient with Prior Encephaloduroarteriosynangiosis

**DOI:** 10.1093/icvts/ivaf246

**Published:** 2025-10-11

**Authors:** Emrah Ereren, Şenay Canikli Adıgüzel, Vaner Köksal, Hüseyin Ağırbaş

**Affiliations:** Department of Cardiovascular Surgery, Samsun University, Faculty of Medicine, Samsun, 55090, Turkey; Department of Anesthesiology and Reanimation, Samsun Training and Research Hospital, Samsun, 55090, Turkey; Department of Neurosurgery, Samsun University, Faculty of Medicine, Samsun, 55090, Turkey; Department of Cardiovascular Surgery, Samsun Training and Research Hospital, Samsun, 55090, Turkey

**Keywords:** Moyamoya disease, coronary artery bypass, carotid artery diseases, cerebral revascularization, encephaloduroarteriosynangiosis, neuroprotection

## Abstract

Background: Moyamoya disease is a progressive steno-occlusive vasculopathy affecting the intracranial internal carotid arteries and posing significant perioperative challenges during cardiac surgery.

Case presentatıon: We present a 54-year-old male with bilateral carotid artery occlusion who had previously undergone left-sided encephaloduroarteriosynangiosis (EDAS), with angiography confirming robust collateral formation from the superficial temporal to middle cerebral artery territory. Three years later, he developed severe multivessel coronary artery disease requiring surgical revascularization. Coronary artery bypass grafting (CABG) was performed using a pump-assisted beating-heart technique without aortic cross-clamping due to heavy ascending aortic calcification. Intraoperative neuroprotection included near-infrared spectroscopy monitoring, controlled PaCO_2_, hematocrit and blood pressure maintenance, mild hypothermia, and avoidance of vasoconstrictors.

Dıscussıon: The patient recovered uneventfully and was discharged without neurological deficits on postoperative day 10. Importantly, unlike previous Moyamoya cases undergoing CABG, this report describes one of the very few documented instances performed after EDAS.

Conclusıon: The presence of established indirect collaterals likely contributed to a favourable neurological outcome, underscoring that staged revascularization may confer significant neuroprotection and should be considered in preoperative planning for selected Moyamoya patients.

## INTRODUCTION

Moyamoya disease (MMD) is a chronic, progressive cerebrovascular disorder characterized by stenosis or occlusion of the intracranial internal carotid arteries and the development of fragile collateral networks at the brain’s base.^1^^,^^2^ Angiographically, MMD presents as fine collateral vessels giving a characteristic “puff-of-smoke” appearance. Clinical presentation ranges from ischaemic or hemorrhagic stroke to transient ischaemic attacks (TIAs), seizures, and cognitive decline.^3^ In patients with concomitant coronary artery disease (CAD), impaired cerebral autoregulation poses a substantial risk during cardiac surgery, particularly with cardiopulmonary bypass (CPB), where systemic hypotension can precipitate cerebral ischaemia.^4^ Indirect cerebral revascularization techniques, such as encephaloduroarteriosynangiosis (EDAS), aim to enhance cerebral perfusion without microvascular anastomosis and have demonstrated efficacy in both paediatric and selected adult populations.^2^^,^^3^ However, their protective role in subsequent CABG procedures is rarely reported. Herein, we present a Moyamoya patient with bilateral internal carotid artery occlusion who underwent unilateral EDAS before multivessel CABG, emphasizing neuroprotective intraoperative strategies and postoperative outcome.

### Case report

**Figure 1.**
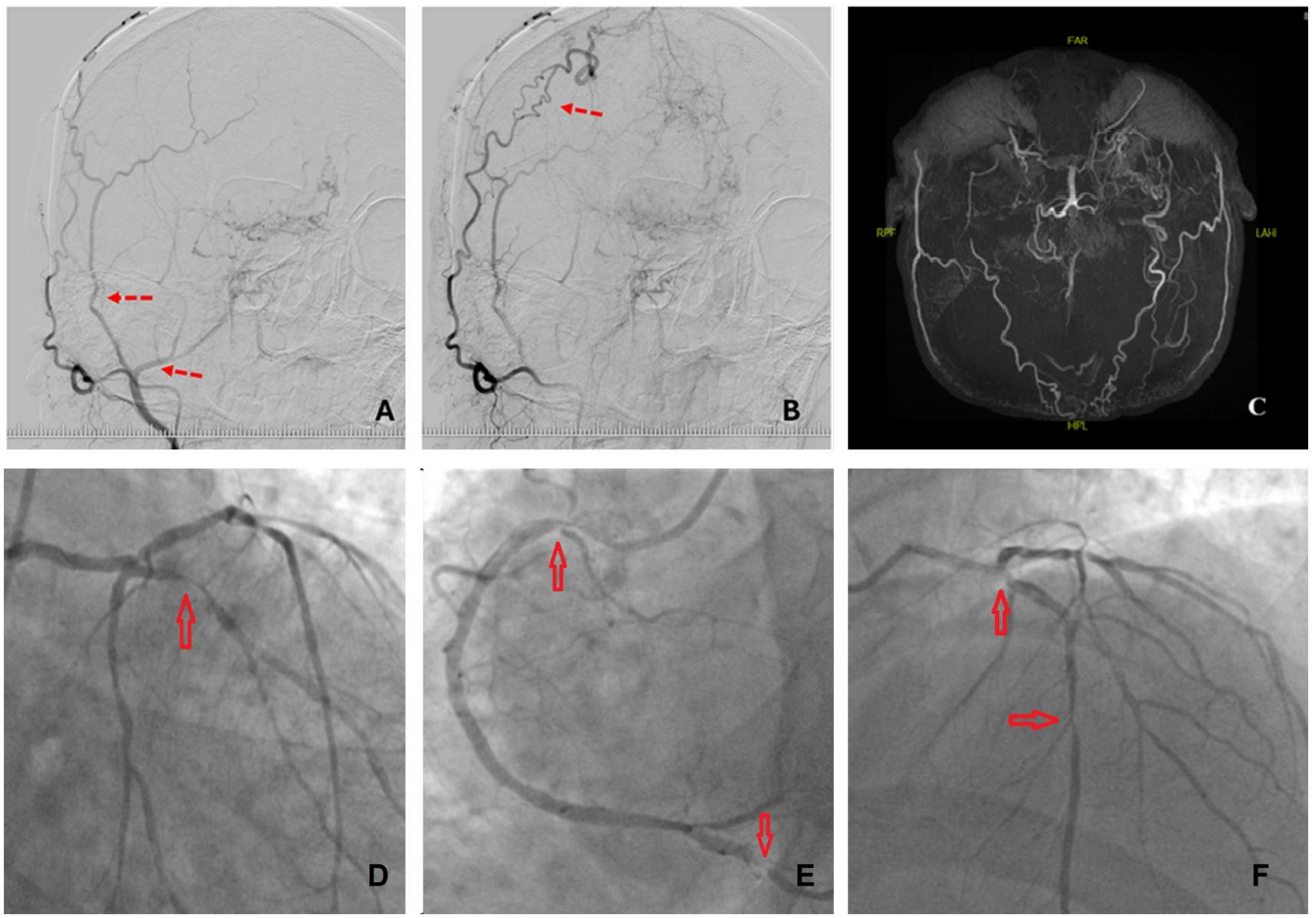
(A) Early Arterial Phase DSA Four Years After Encephaloduroarteriosynangiosis. (B) Delayed Phase Demonstrating Robust Collateral Perfusion of the MCA Territory. (C) Preoperative MR Angiography Showing Bilateral ICA Occlusion with “Puff-of-Smoke” Collaterals. (D) Left Coronary Angiography Showing Mid/Distal Circumflex Stenosis (70%) and (E) sequential LAD Lesions (50% Ostial, 90% Mid). (F) Right Coronary Angiography Demonstrating Tight Proximal Stenosis and Severe PDA Narrowing (90%). Findings İndicated Three-Vessel Disease Requiring Surgical Revascularization.

A 54-year-old male with MMD, hypertension, and diabetes mellitus presented with recurrent angina. Echocardiography revealed preserved left ventricular ejection fraction (55%) and mild valvular regurgitation. Coronary angiography demonstrated severe three-vessel disease involving the left anterior descending (LAD), first diagonal, circumflex, and right coronary arteries (Figure 1D–F). In 2021, the patient had undergone left-sided EDAS, in which the superficial temporal artery and attached temporal muscle were transposed onto the brain surface targeting the middle cerebral artery (MCA) territory. Postoperative imaging confirmed robust STA-to-MCA collateralization (Figure 1A–C). During the 3 years following EDAS, the patient remained neurologically stable without recurrent ischaemic events. Carotid Doppler revealed complete right ICA occlusion and near-total left ICA occlusion. Preoperative assessment identified calcified radial arteries, elevated body mass ındex (BMI) (31 kg/m^2^), and poorly controlled diabetes (HbA1c: 13.2%), precluding the use of bilateral internal mammary arteries. Left Internal Mammary Artery (LIMA) and saphenous vein grafts were chosen. The patient was classified as ASA III. Intraoperative monitoring included ECG, invasive arterial pressure, bispectral index (BIS), and near-infrared spectroscopy (NIRS). Cerebral oxygen saturation was maintained at ≥80% of baseline, with PaCO_2_ held at 40–45 mmHg, systolic BP >100 mmHg, and hematocrit >21%. Vasoconstrictors were avoided. Haemodynamic stability was supported with esmolol and nicardipine. Hypotension during graft positioning prompted initiation of CPB. Median sternotomy was performed. LIMA was anastomosed to the LAD on the beating heart. Arterial cannulation was established proximal to the brachiocephalic trunk due to aortic calcification, and cross-clamping was avoided to minimize embolic risk. Saphenous vein grafts were connected to OM1 and PDA using a pump-assisted beating heart technique. CPB time was 74 min; total operative time was 320 min. The patient was extubated 10 hours postoperatively without neurological deficits, mobilized by postoperative day 3, and discharged home on day 10.

## DISCUSSION

Moyamoya disease patients undergoing cardiac surgery face increased risk of perioperative cerebral ischaemia due to compromised perfusion and limited collateral capacity.^1^^,^^5^ Neuroprotective strategies, including continuous NIRS monitoring, maintenance of normocapnia, mild hypothermia, and strict blood pressure control without vasoconstrictors, are essential in mitigating this risk. In this case, cerebral oximetry remained stable throughout surgery, and the patient had no postoperative neurological complications, consistent with literature supporting real-time cerebral monitoring.^4^^,^^5^ A unique aspect was the stabilizing effect of prior EDAS in a patient with bilateral ICA occlusion. Encephaloduroarteriosynangiosis is a recognized technique to augment perfusion through angiogenesis,^3^ but reports on its protective role during subsequent cardiac surgery are scarce. Robust collateral flow from the STA to MCA likely contributed to the favourable outcome. Indirect revascularization techniques such as EDAS typically require several months to achieve optimal angiogenesis, but once mature, they may sustain adequate perfusion during systemic stress. Various surgical strategies for MMD patients with CAD include off-pump CABG, pump-assisted beating heart surgery, intra-aortic balloon pump support, and minimally invasive direct CABG (MIDCAB).^1^^,^^4^^,^^5^ The choice depends on coronary anatomy, comorbidities, and conduit quality. In this patient, high BMI, calcified radial arteries, and diffuse CAD favoured median sternotomy with a pump-assisted approach, avoiding cross-clamping to reduce embolic risk.

### Haemodynamic management and literature support

In patients with Moyamoya disease undergoing CABG, perioperative haemodynamic management requires particular attention to the balance between systemic stability and cerebral perfusion. Our strategy of avoiding vasoconstrictors and using esmolol and nicardipine for blood pressure modulation aligns with previously reported approaches emphasizing the importance of normocapnia, mild hypothermia, and hematocrit preservation. Recent reports have highlighted that alternative vasopressor strategies—such as low-dose norepinephrine or vasopressin—may be preferable to phenylephrine, which could exacerbate cerebral hypoperfusion by increasing vascular resistance.^6–8^ These considerations underscore the importance of individualized haemodynamic support protocols in Moyamoya patients requiring cardiac surgery.

### Long-term outcomes and neuroprotection

Although our patient had a favourable short-term postoperative course, long-term outcomes regarding cerebral perfusion and collateral sustainability remain underreported in the literature. A few small case series suggest that prior indirect cerebral revascularization with EDAS or EMS may provide durable neuroprotection during systemic stress, but prospective data are lacking.^9^^,^^10^ Further studies with extended follow-up are necessary to evaluate whether the angiogenesis induced by EDAS confers a sustained benefit in the setting of major cardiac interventions.

### Expanding evidence and comparative approaches

Recent literature (post-2020) has described the use of advanced monitoring modalities, hybrid revascularization strategies, and minimally invasive CABG approaches in Moyamoya patients. While MIDCAB and off-pump techniques have been successfully reported in selected cases, our patient’s anatomy and comorbidities favorued a pump-assisted beating-heart approach. The expanding evidence base highlights that surgical decision-making in this context should remain highly individualized, taking into account cerebral perfusion status, collateral maturity, and conduit quality.^9^ To further contextualize our findings, we provide a summary of preclinical and clinical reports evaluating the protective role of EDAS in Moyamoya patients undergoing CABG (Table 1).

**Table 1.** Evidence Referencing Indirect Cerebral Revascularization (EDAS/EMS) and Outcomes around Coronary Surgery

Author/year	Study type	Population/model	Surgical procedure	Neuroprotective outcome
Komiyama et al. (2003)	Case report	Adult, Moyamoya + CAD	MIDCAB	No neurological deficit
Haberal et al. (2013)	Case report	Adult, Moyamoya + CAD	On-pump CABG	Uneventful recovery
Köksal et al. (2015)	Case report	Paediatric, Moyamoya	EMS procedure only	Robust angiogenesis, stable outcome
Küçükosman et al. (2018)	Case report	Adult, Moyamoya + CAD	Off-pump CABG with NIRS	Stable cerebral oximetry
Current Case (2025)	Case report	Adult, Moyamoya + CAD	Pump-assisted beating heart CABG post-EDAS	No neurological deficit

Note: Previous Moyamoya cases undergoing CABG (Komiyama, Haberal, Küçükosman) did not involve prior EDAS/EMS. Our present case is among the very few documented instances of CABG performed after indirect cerebral revascularization.

Abbreviations: CAD, coronary artery disease; CABG, coronary artery bypass grafting; MIDCAB, minimally invasive direct coronary artery bypass; NIRS, near-infrared spectroscopy; EMS, encephalo-myo-synangiosis; EDAS, encephaloduroarteriosynangiosis.

Importantly, previously reported Moyamoya cases undergoing CABG did not involve prior indirect cerebral revascularization. Our case, therefore, represents one of the very few documented instances of CABG performed after EDAS, supporting the concept that established indirect collaterals may confer significant neuroprotection during high-risk cardiac surgery and should be considered in preoperative planning for selected MMD patients.

### Limitations

This report has several limitations. First, it represents a single case and therefore cannot be generalized to all Moyamoya patients undergoing CABG. Second, prospective or controlled data are not available to establish a causal relationship between prior EDAS and the favourable neurological outcome observed. Third, the protective effect of EDAS could not be directly quantified, as collateral circulation and cerebral perfusion were assessed only through imaging and intraoperative monitoring. Finally, the scarcity of comparable cases in the literature limits the strength of conclusions regarding long-term neuroprotection. Despite these limitations, our case adds to the growing body of evidence and may encourage further systematic investigations.

## CONCLUSION

Unilateral EDAS can provide effective neuroprotection during complex cardiac surgery in Moyamoya patients with bilateral carotid occlusion. Meticulous preoperative assessment, tailored intraoperative strategies, and real-time cerebral monitoring enabled successful multivessel CABG without neurological sequelae in this high-risk patient. Importantly, this case represents one of the very few documented instances of CABG performed after EDAS, supporting the concept that established indirect collaterals may confer significant neuroprotection and should be considered in preoperative planning for selected Moyamoya patients.

## FUNDİNG

No specific funding was received for this study.

## CONFLICT OF INTEREST

The authors collectively declare that there are no conflicts of interest regarding the publication of this paper.

## DATA AVAILABILITY

The data underlying this article are available in the article and in its online supplementary material.

## ETHICAL STATEMENT

This case report was conducted in accordance with the principles of the Declaration of Helsinki. Ethical approval was not required for this case report according to institutional policy. Written informed consent was obtained from the patient for publication.
